# Editorial of Special Issue “Functional Nanomaterials Based on Self-Assembly”

**DOI:** 10.3390/nano13233062

**Published:** 2023-12-01

**Authors:** Pavel Padnya

**Affiliations:** A.M. Butlerov’ Chemistry Institute, Kazan Federal University, 18 Kremlevskaya Street, 420008 Kazan, Russia; padnya.ksu@gmail.com

In recent years, the design and creation of new functional nanosystems and nanomaterials similar in their properties to biological systems showed remarkable progress as an interdisciplinary field of research combining chemistry, biology, and physics [1]. The creation of such nanomaterials can be based on both organic and inorganic components, e.g., synthetic molecules (small ones and macrocyclic molecules), nanostructures (nanoparticles and organic-inorganic complexes), and natural polymers (nucleic acids, proteins, and carbohydrates). This Special Issue covers a wide range of research topics related to the design and application of soft and hard functional nanomaterials.

It is well known that self-assembly is often used to create nanomaterials and composites to modify the sensor interface. This often leads to improvements in their properties, e.g., efficiency and selectivity of the detection of various analytes. The work by Prof. Evtugyn et al. [2] is an example of the creation of a voltammetric sensor for the detection of anticancer drug doxorubicin (DOX). The authors used a DNA-polyphenothiazine composite as a modifier able to recognize, accumulate and detect target species. Such hybrid materials exert unique characteristics to achieve the DOX determination in the concentration range from 10 pM to 0.2 mM (limit of detection 5 pM). 

A review article by Prof. Bourgault and colleagues [3] is devoted to the latest achievements in development of functional systems to be obtained by self-assembly of π-conjugated systems on the perylene diimide platform (PDI). These systems show unique optical and electronic properties and demonstrate chemical, thermal and photo-stability. The synthesis, self-assembly, and applications of the PDI bioconjugates with biological molecules such as oligonucleotides and peptides are discussed in detail. The presented relationships between the structure of the PDI derivatives and their properties contribute to the tunable design and creation of innovative functional biomaterials demanded in various biomedical and nanotechnological applications. 

In recent decades, the chemistry of synthetic macrocyclic compounds such as (thia)calixarenes and pillararenes has been actively developed. The group of Prof. Stoikov is one of the leaders in this field in Russia. In recent work presented by this research team, Shurpik et al. [4] showed that decasubstituted thiolated pillar[5]arenes were able to form polymer self-healing films via thiol/disulfide redox cross-linking. The addition of the antimicrobial drug moxifloxacin to these films resulted in a marked inhibition of biofilm formation of *Staphylococcus aureus* and *Klebsiella pneumonia* bacteria. In another work by Prof. Stoikov [5], monosubstituted pillar[5]arene derivatives containing a diethylenetriamine spacer with one or two terminal carboxyl groups were synthesized. Solid lipid nanoparticles (SLNs) were assembled based on these macrocyclic compounds. The authors showed that the number of carboxyl groups in the macrocycle dramatically affected the size and shape of the yielding SLNs. In the case of the macrocycles with one carboxyl group, stick-like structures with the diameter of 50–80 nm and the length of 700–1000 nm were formed. The obtained functional systems based on the pillar[5]arenes can be further used to create promising materials. 

A new approach to noninvasive tumor cell therapy was proposed by the scientific team of Prof. Abakumov [6]. The authors created a novel genetically encoded material based on *Quasibacillus thermotolerans* encapsulin stably expressed in mouse 4T1 breast carcinoma cells. The material structure contained the enzyme ferroxidase that oxidized Fe(II) ions to Fe(III) to form iron oxide nanoparticles. The authors showed that the expression of transgenic sequences did not affect cell viability, and the presence of magnetic nanoparticles resulted in an increase in T2 relaxivity. 

Prof. Fery’s group [7] synthesized gold nanorods via bottom-up template-assisted self-assembly. The authors studied the conductivity of the obtained nanomaterials and found that multiple parallel AuNR lines (>11) were required to achieve predictable conductivity properties. The results can be used to create resistance-based sensor wires or as anisotropically conducting surfaces in devices. 

A new photoactivatable antibacterial drug based on gold nanoparticles (~50 nm) coated with polyethylene glycol was proposed and obtained by Prof. La Deda and co-workers [8]. The resulting supramolecular systems showed a 46% growth inhibition in the dark and a 99% growth inhibition in the light. The photothermal effect was shown to be due to the formation of a light-induced cluster of gold nanoparticles (at low concentrations). The authors suggested that the bacterium wall catalyzed the formation of these clusters that were increased in the measured temperature and caused bactericidal effect. 

Prof. Woo and colleagues [9] studied the self-assembly of model arylate polyester polymers with different numbers of methylene segments (n = 3, 9, 10, and 12) in solid state. These polymers crystallized depending on the conditions as similar types of periodically banded spherulites. The observed phenomena were explained on the basis of crystal-by-crystal self-assembly with periodic branching and discontinuous intersection. The obtained results facilitate better understanding of mechanisms of periodic phenomena in crystal assembly in the scientific community. 

Vasilieva et al. [10] studied the self-assembly of surface-active amphiphilic compounds containing different cationic head groups (pyrrolidinium and imidazolium) with anionic structures, i.e., polyacrylic acid and human serum albumin. The addition of polyanions led to a two-order decrease in the critical concentration of surfactant aggregation (from 1000 µM to 10 µM). As a result, nanocontainers capable of efficient drug loading (meloxicam, warfarin, and amphotericin B) were created based on the obtained supramolecular systems. 

Another example of the use of self-assembly to create nanomaterials is the work of Huang and colleagues [11] devoted to the creation of star copolymers with a defined number of necessary monomer links. The authors obtained nanostructures of about 15 nm in size with low polydispersity. Thus, this work is an example of successful post-modification of linear polymers to obtain materials with new properties. 

Gileva et al. [12] proposed and developed a novel system for targeted delivery of DOX based on polyelectrolyte multicellular capsules modified with the DR5-B protein. The developed supramolecular systems showed improved antitumor activity due to high tumor targeting and the synergistic effect of DR5-B and DOX. 

Overall, this Special Issue includes 11 excellent papers of the contributors from eight countries on the design, synthesis and application of self-assembled functional nanomaterials and covers various fields of nanotechnology, chemistry, biology and material science. As guest editor of Special Issue “Functional Nanomaterials Based on Self-Assembly”, I thank all authors, reviewers, and assistant editors for their valuable contributions. I hope that the publications presented in this Special Issue will be interesting and useful to readers. 
